# Extension-Type Spinal Fractures on Flat Surfaces: Forensic Autopsy Findings From Two Cases

**DOI:** 10.7759/cureus.89034

**Published:** 2025-07-30

**Authors:** Ikuto Takeuchi, Motoo Yoshimiya, Atsushi Ueda, Yu Kakimoto

**Affiliations:** 1 Department of Forensic Medicine, Tokai University School of Medicine, Isehara, JPN

**Keywords:** cardiopulmonary resuscitation, extension-distraction injury, forensic autopsy, spinal fracture, thoracic kyphosis

## Abstract

Extension-distraction injuries of the spine, classified as B3 in the AOSpine Trauma Classification system, are generally attributed to hyperextension mechanisms. However, under specific biomechanical conditions, such injuries may occur even on flat surfaces not typically associated with high-energy trauma. We present two forensic autopsy cases of extension-type vertebral fractures occurring in flat-surface settings. The first case involved a man in his 50s who sustained a thoracic vertebral fracture and cardiac rupture after being compressed dorsally in the prone position by a 1,000-kg brush cutter. The second case involved a man in his 90s with kyphosis who sustained a lumbar extension-type fracture during cardiopulmonary resuscitation after collapsing outdoors. These cases highlight the importance of considering posture and biomechanical forces in the forensic evaluation of spinal fractures.

## Introduction

Spinal fractures are commonly classified using the AOSpine Trauma Classification system. Among these, extension-type injuries are designated as B3-type fractures, which are relatively uncommon and characterized by anterior distraction (pulling apart) of the vertebral body [1]. Such injuries often result in marked instability and frequently require surgical stabilization [2,3].

Extension-type fractures typically occur in contexts involving apparent hyperextension of the torso, such as in vehicular collisions or falls. However, similar patterns may also develop in flat-surface environments without obvious hyperextension, depending on specific body postures and directions of force. Understanding these mechanisms requires consideration of physiological spinal curvature and the progression of age-related kyphosis [4,5], which may fix the spine in a flexed posture, making it more vulnerable to extension forces when external pressure is applied. These injuries are typically identified through postmortem CT imaging or autopsy by detecting anterior widening of the vertebral body and associated soft tissue disruption.

We present two cases of extension-type spinal fractures that occurred without any overt hyperextension events. Both were associated with flat-surface scenarios and highlight how particular postural and biomechanical conditions can produce these injuries. We discuss their forensic implications and the underlying mechanical context. Misunderstanding these injuries may lead to false suspicion of abuse or incorrect cardiopulmonary resuscitation (CPR), underscoring the need for careful forensic evaluation.

## Case presentation

Case 1

A man in his 90s with no known medical history was found collapsed outdoors. Dashcam footage from a nearby vehicle showed signs of distress, followed by a gradual transition into a lateral decubitus position, after which he became motionless. Emergency responders found him in cardiac arrest. He received CPR, including sternal compressions, during transport, but was pronounced dead upon hospital arrival.

**Figure 1:**
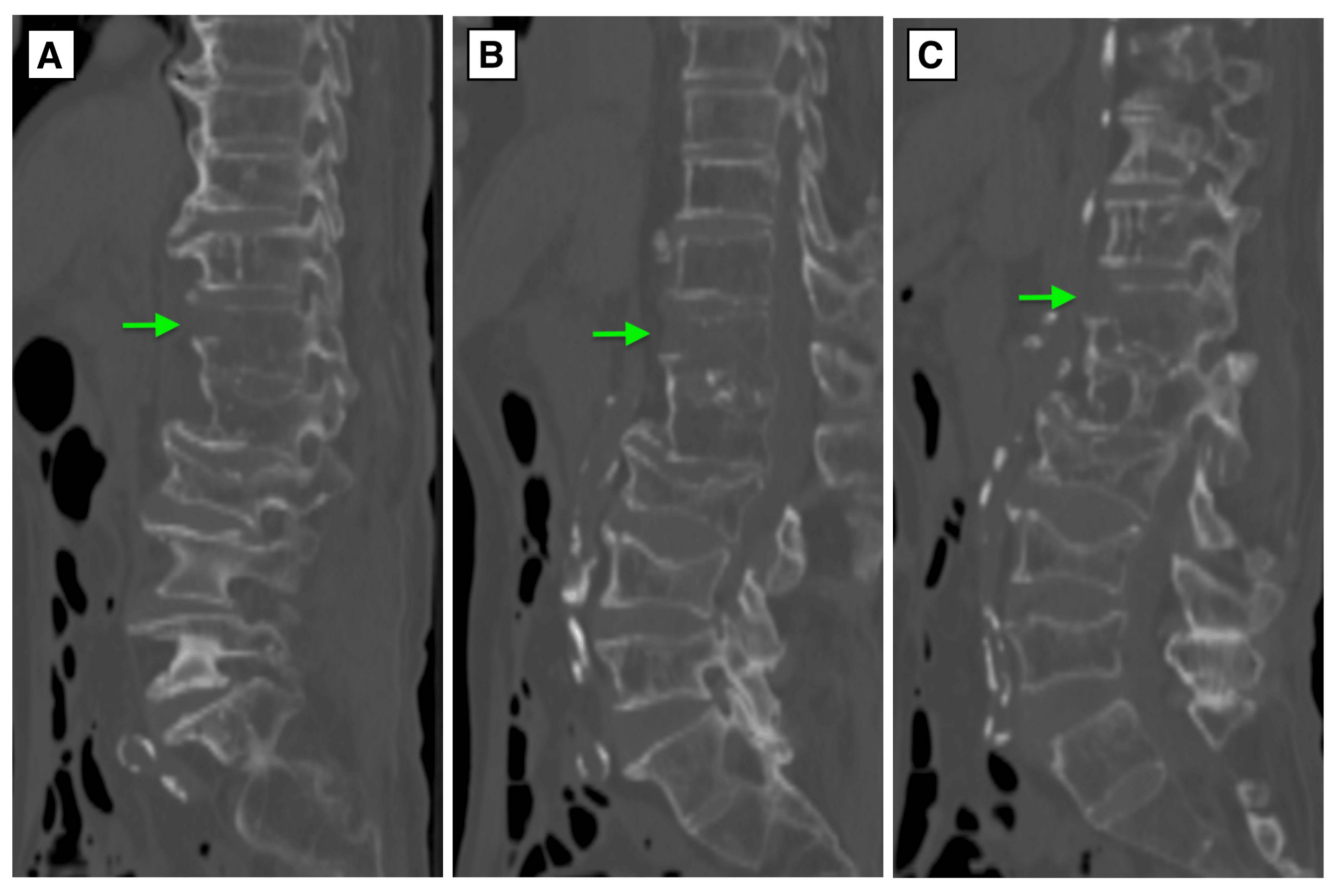
Sagittal CT images of the first lumbar vertebra (Case 1). (A) Right pedicle level. The arrow indicates the fracture site. (B) Midline. The arrow indicates the fracture site. (C) Left pedicle level. The arrow indicates the fracture site.

**Figure 2:**
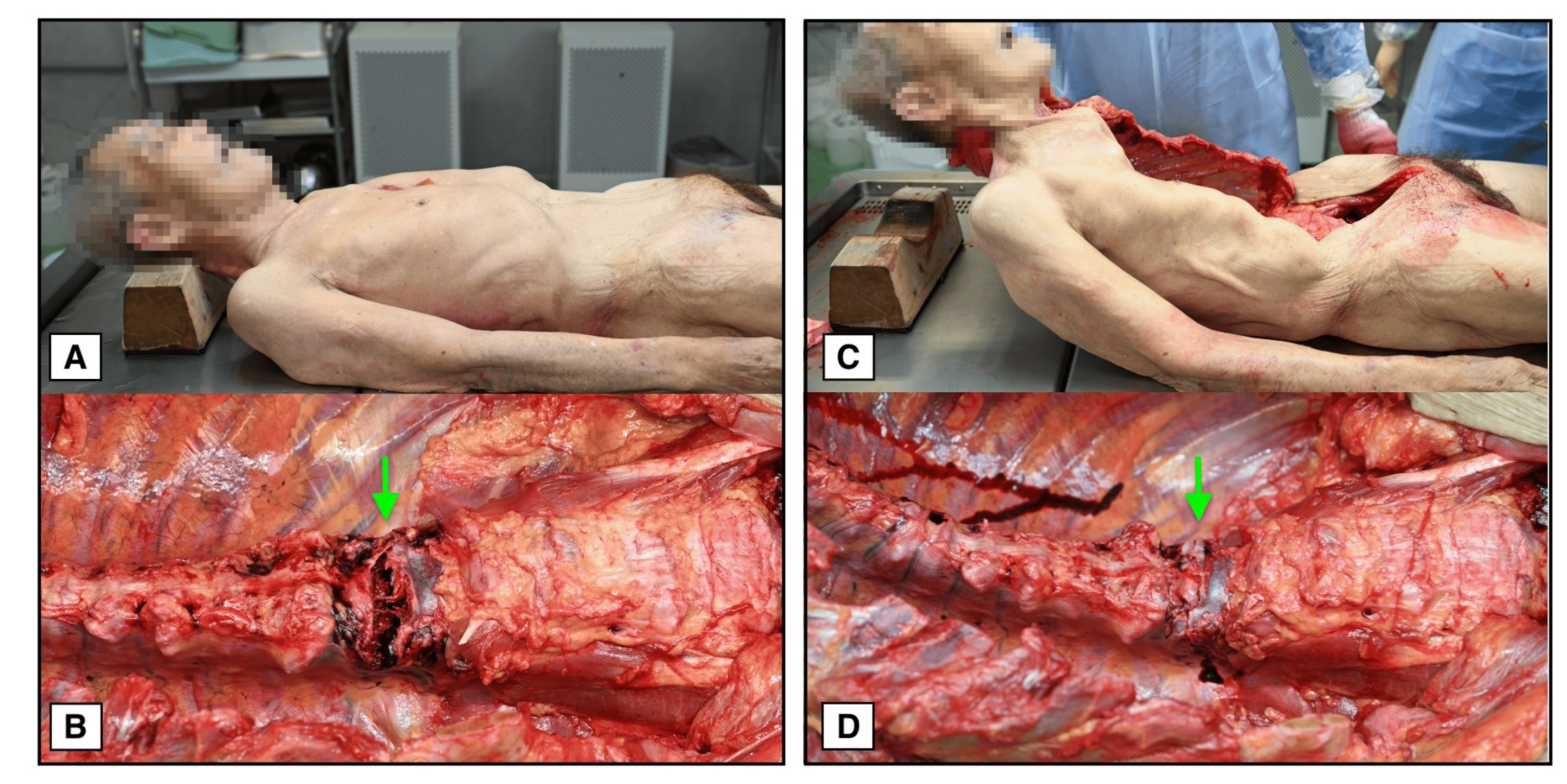
Gross autopsy images of the first lumbar vertebra (Case 1). (A) Observation in the supine position showing anterior distraction of the L1 vertebral body. (B) Observation in a recreated kyphotic posture showing normal alignment of the vertebral body without compression. Arrows indicate the fracture site in both images.

A forensic autopsy revealed ischemic heart disease as the cause of death. No other traumatic injuries were identified. However, postmortem CT (Figures 1A-1C) and autopsy (Figure 2) identified an extension-type fracture of the first lumbar vertebra.

A police investigation confirmed a history of kyphosis. Toxicological screening, including alcohol and drug testing, returned negative results.

Case 2

**Figure 3:**
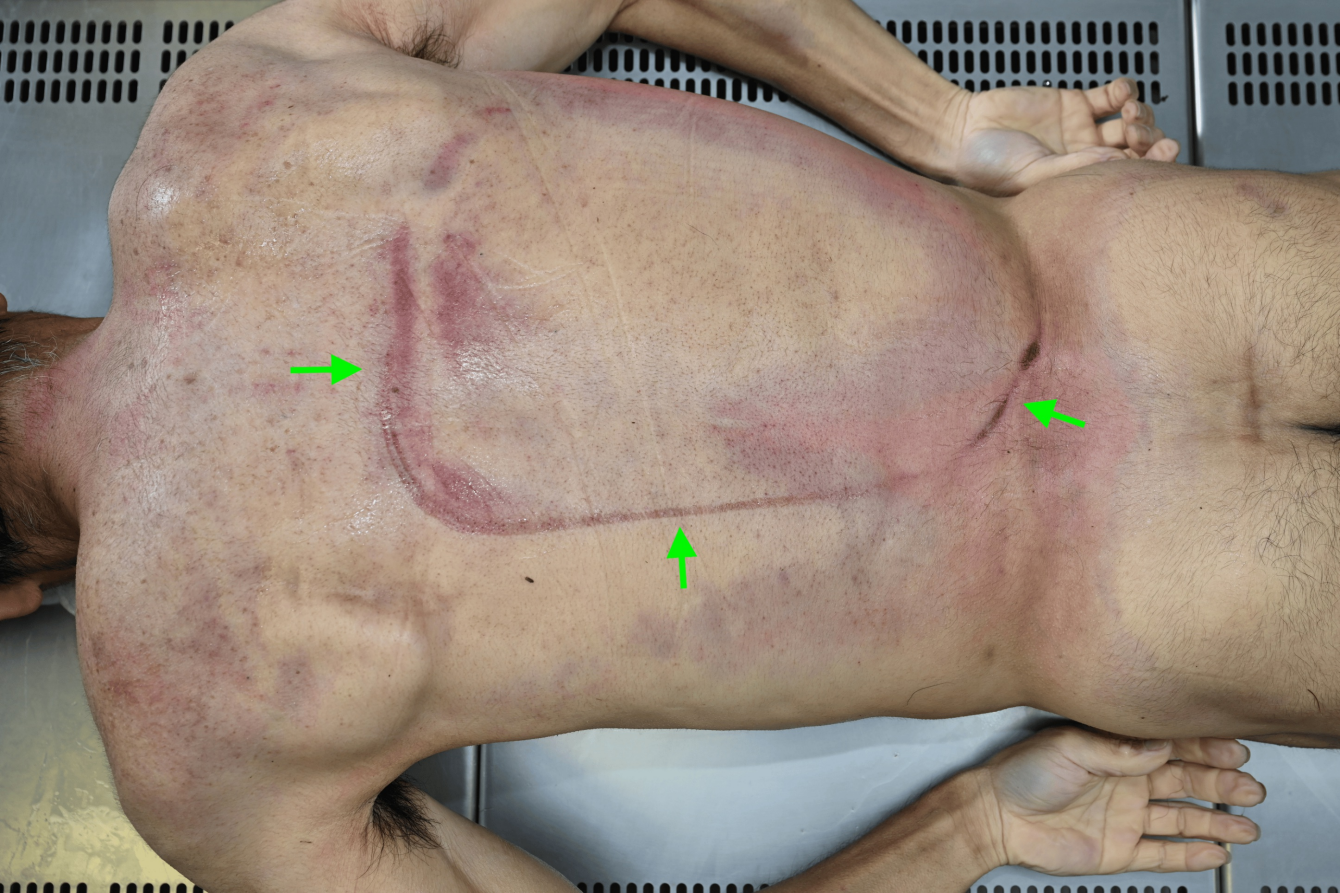
Posterior compression marks on the back (Case 2). External examination revealing reddish-purple contusions on the back, presumed to result from brush cutter compression. The arrow indicates the area of impact.

A man in his 50s with no known medical history was operating a 1,000-kg brush cutter on a slope when the machine overturned. He was ejected and found prone, with the machine presumably resting on his back. He was in cardiac arrest when discovered, and CPR was initiated by emergency responders, but was unsuccessful. He was pronounced dead upon hospital arrival. A forensic autopsy revealed distinct compression marks on the back (Figure 3).

**Figure 4:**
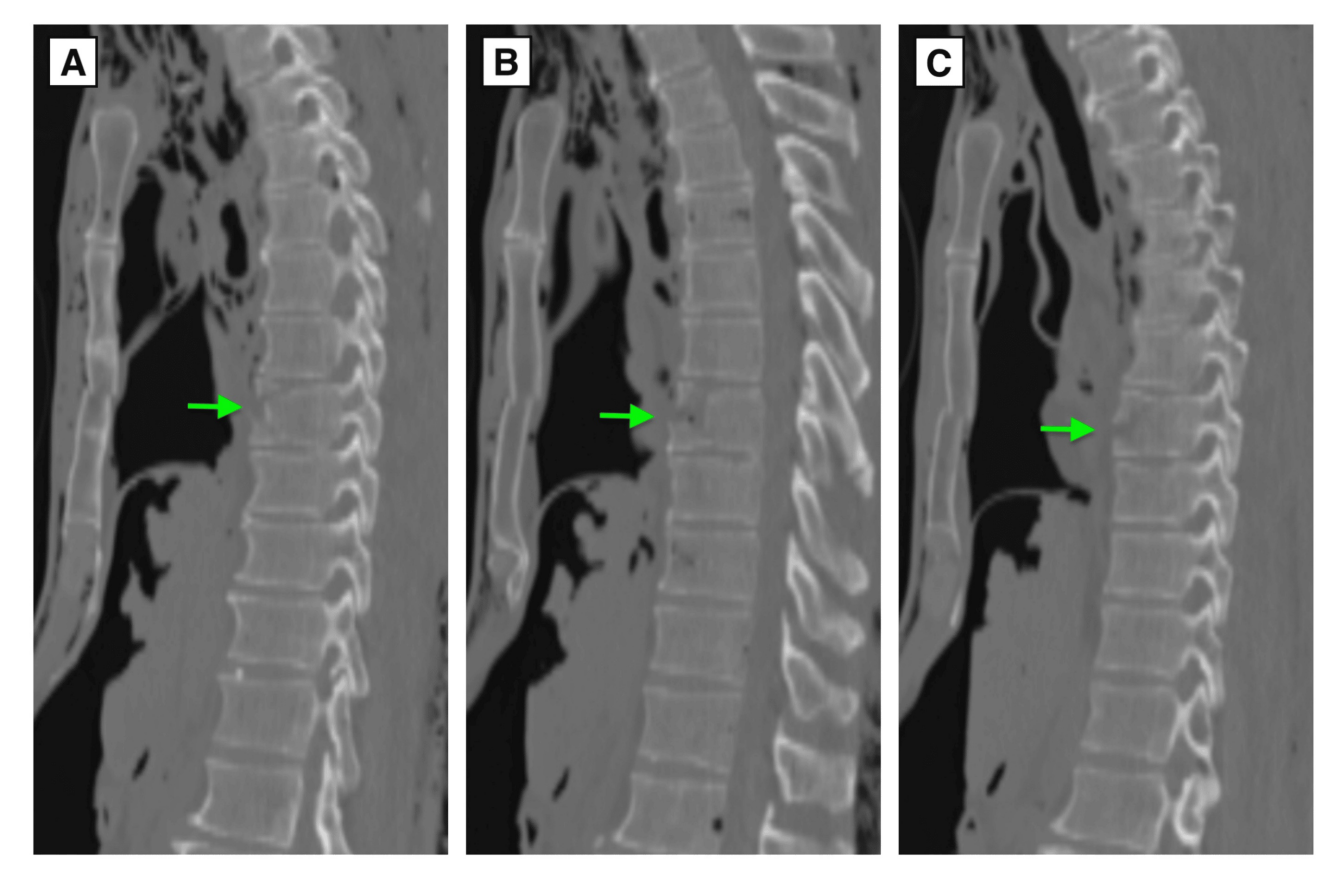
Sagittal CT images of the sixth thoracic vertebra (Case 2). (A) Right pedicle level. The arrow indicates the fracture site. (B) Midline. The arrow indicates the fracture site. (C) Left pedicle level. The arrow indicates the fracture site.

**Figure 5:**
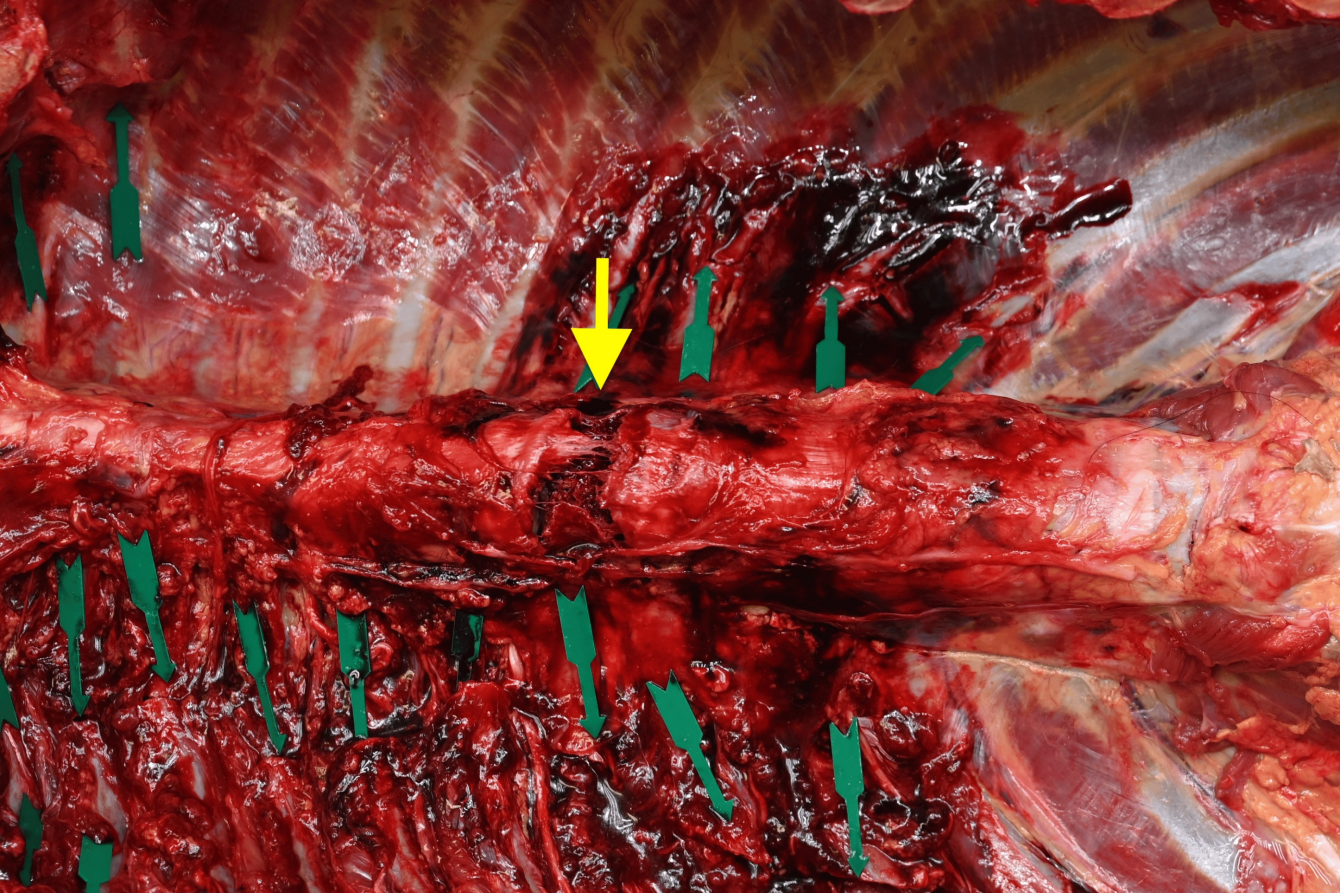
Gross autopsy image of the sixth thoracic vertebra (Case 2). Observation in the supine position shows anterior distraction of the T6 vertebral body. The yellow arrow indicates the fracture site.

Postmortem CT (Figures 4A-4C) and autopsy (Figure 5) showed an extension-type fracture of the sixth thoracic vertebra.

Additionally, right-dominant bilateral multiple rib fractures, traumatic hemopneumothorax, and cardiac rupture were observed. Based on the comprehensive assessment of these findings, cardiac rupture was determined to be the fatal injury. As evident in Figure 4, the T6 vertebra is located directly posterior to the sternum. It was inferred that the structural failure of the thoracic arch allowed direct external force to impact the heart, resulting in cardiac rupture. Toxicological screening, including alcohol and drug testing, returned negative results.

## Discussion

These cases suggest that extension-type spinal fractures can occur even on flat surfaces. The presence of physiological spinal curvature [4] and age-related kyphosis [5] likely contributed to the development of these fractures.

**Figure 6:**
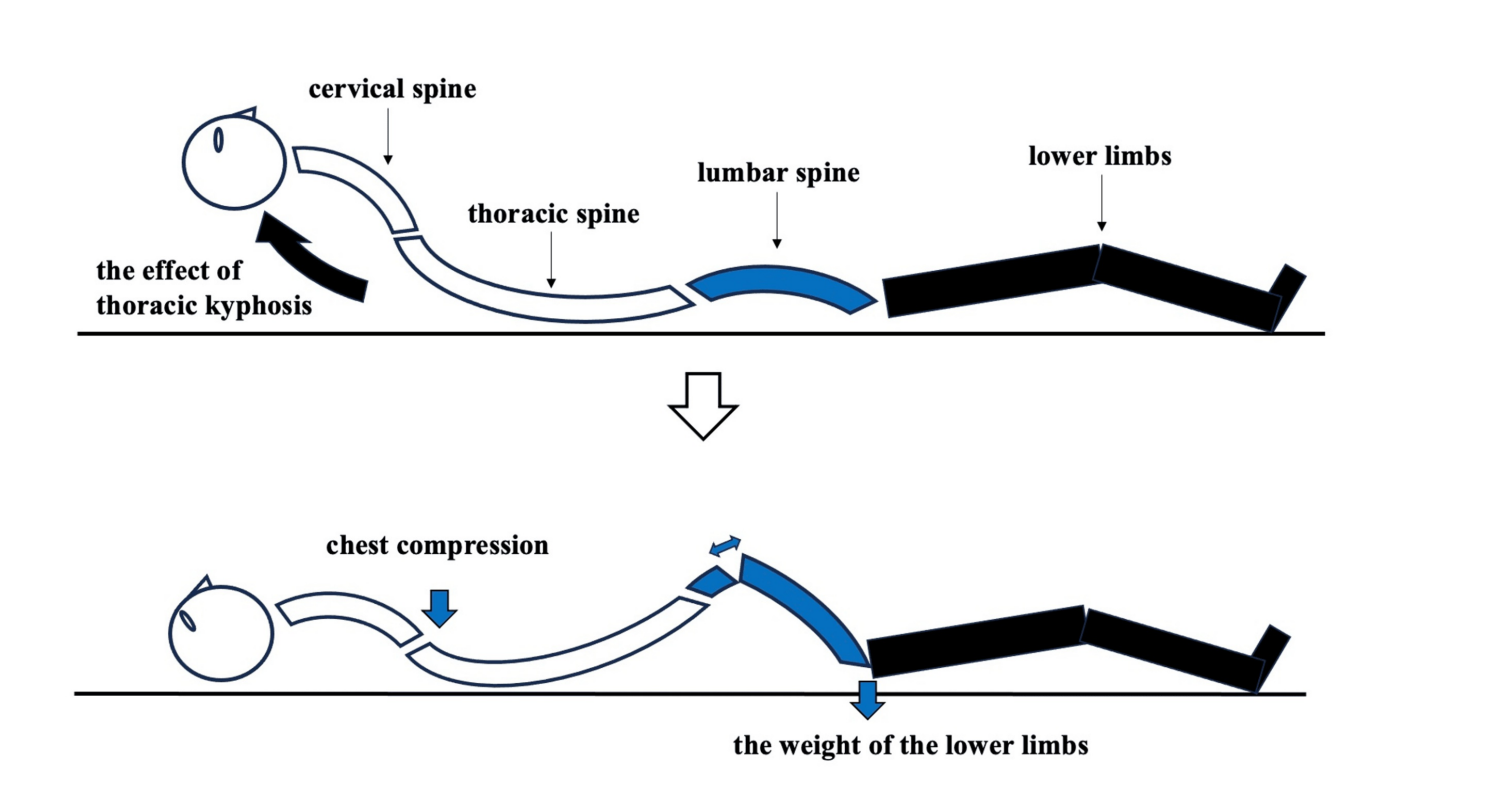
Lumbar hyperextension during chest compression in the supine position (Case 1). A schematic diagram depicting the mechanism of an extension-type lumbar fracture in Case 1. Hyperextension force was generated by sternal chest compressions combined with the downward weight of the lower limbs in a kyphotic spine fixed in the supine position. Arrows indicate the direction of the external force applied. The illustration was created by the authors based on standard anatomical knowledge.

In Case 1, chest compressions were applied in the supine position to a patient with a preexisting kyphotic deformity. The fixed body posture likely generated a relative hyperextension force on the lumbar spine, resulting in an extension-type fracture (Figure 6).

Dashcam footage confirmed a slow progression into the lateral decubitus position without a fall or abrupt motion, ruling out any obvious external trauma before resuscitation. Given the timing of the injury, fracture morphology, and lack of other traumatic events, a spontaneous fracture appears unlikely, and the injury is most plausibly attributed to chest compressions during CPR. In the context of a kyphotic spine, the supine position during CPR likely imposed an abnormal extension vector on the lumbar spine, as the thoracic cage and pelvis remained fixed while compressive force was applied anteriorly. This mechanical setup could have induced anterior distraction at the L1 vertebra. Although the patient was elderly and may have had reduced bone mineral density, the injury characteristics and their temporal association with chest compressions support a traumatic rather than spontaneous osteoporotic origin.

Various complications of chest compressions have been documented [6], including thoracic extension-type fractures following CPR [7]. Similar fractures have been observed during resuscitation in elderly patients with kyphosis [8]. Extension-type injuries are also common in individuals with ankylotic spinal disorders [9]. In aging populations, understanding these mechanisms is essential not only in forensic medicine but also in clinical and emergency care.

**Figure 7:**
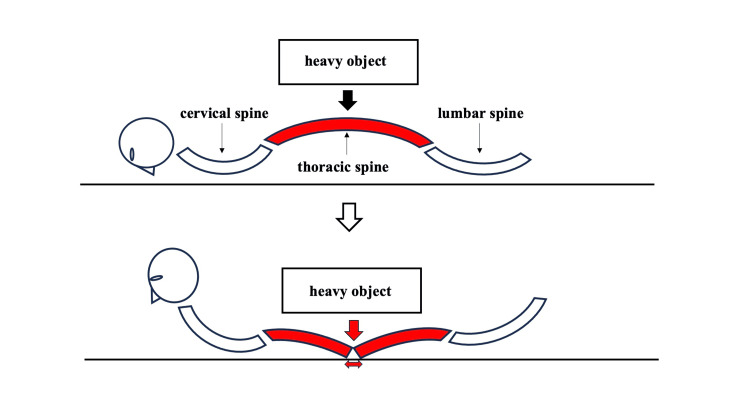
Thoracic hyperextension due to dorsal compression in the prone position (Case 2). A schematic diagram showing the mechanism of an extension-type thoracic fracture in Case 2. Dorsal compression in the prone position forced the physiological curvature of the thoracic spine into hyperextension, resulting in anterior vertebral distraction. Arrows indicate the direction of external force applied. The illustration was created by the authors based on standard anatomical knowledge.

In Case 2, a prone body position combined with direct dorsal compression by a heavy object likely forced the thoracic spine into hyperextension, causing a fracture (Figure 7).

The rib fractures in Case 2 were predominantly located on the right side and posterior aspects, corresponding to the area of direct compression by the overturned brush cutter. While CPR was performed, the distribution of fractures was inconsistent with typical resuscitation-related injuries, which usually involve the anterior chest. Therefore, the rib fractures were most likely caused by the external mechanical force rather than by chest compressions. This injury pattern suggests substantial dorsal compression, which likely contributed to the hyperextension mechanism responsible for the thoracic vertebral fracture. Although spinal fractures due to dorsal compression have been reported [10], we found no prior cases describing thoracic extension-type fractures caused by prone-position compression on flat surfaces. This case, thus, represents a rare and atypical injury pattern occurring under unique biomechanical conditions.

While neither case involved traditional high-energy trauma, from a spinal biomechanics perspective, it is plausible that fixed postures with spinal curvature subjected to external forces can result in hyperextension-type fractures. Fracture morphology is closely linked to the direction and manner of force application [11]. In spinal trauma, fracture patterns often provide insights into the mechanism of injury [12]. In both cases, detailed documentation of the circumstances and resuscitative efforts enabled reasonable reconstruction of the trauma mechanisms.

However, because extension-type fractures are often associated with high-energy mechanisms, their presence could be misinterpreted as evidence of assault or falls if contextual information is lacking. Therefore, recognizing that extension-type fractures may occur under specific biomechanical conditions, even in flat-surface environments, is critical for accurate forensic diagnosis and trauma reconstruction. Furthermore, extension-type fractures are highly unstable and often require surgical intervention [2,3]. Unlike other types of spinal fractures, it has been reported that supine immobilization may fail to stabilize these injuries and could exacerbate hemorrhage [13]. Therefore, in clinical cases where the patient survives, rapid recognition and appropriate management of these injuries are essential to prevent secondary complications such as neurological deficits or delayed spinal instability.

This report is limited by the small number of cases and the lack of bone density or histological analysis. The retrospective nature of forensic autopsy also limits available clinical information. These factors may affect the interpretation of the findings.

## Conclusions

Extension-type spinal fractures can occur not only in classical high-energy hyperextension trauma but also under specific postural and biomechanical conditions, even on flat surfaces. In forensic practice, it is important to recognize that these fractures do not necessarily indicate third-party involvement or falls. Accurate interpretation of such findings is essential for determining the cause and mechanism of injury. In clinical settings, extension-type fractures are highly unstable and may not be suitable for conservative management. Prompt diagnosis and appropriate treatment are crucial to prevent secondary complications, such as neurological deficits or delayed spinal instability. Understanding the mechanisms underlying these injuries, as illustrated in the present cases, is vital for both forensic assessment and clinical decision-making. Additionally, these cases may offer insights applicable to broader clinical settings, particularly when managing elderly or kyphotic patients under mechanical stress during resuscitation or positioning.
